# Two decades of non-invasive genetic monitoring of the grey wolves recolonizing the Alps support very limited dog introgression

**DOI:** 10.1038/s41598-018-37331-x

**Published:** 2019-01-16

**Authors:** Christophe Dufresnes, Nadège Remollino, Céline Stoffel, Ralph Manz, Jean-Marc Weber, Luca Fumagalli

**Affiliations:** 1 Laboratory for Conservation Biology, Department of Ecology and Evolution, Biophore Building, University of Lausanne, CH-1015 Lausanne, Switzerland; 2 Department of Animal & Plant Sciences, University of Sheffield, Alfred Denny Building, Western Bank, Sheffield, S10 2TN United Kingdom; 3 KORA, Carnivore Ecology and Wildlife Management, Thunstrasse 31, CH-3074 Muri, Switzerland; 4 Fauna Découverte, Pré-Girard 18, CH-2067 Chaumont, Switzerland

## Abstract

Potential hybridization between wolves and dogs has fueled the sensitive conservation and political debate underlying the recovery of the grey wolf throughout Europe. Here we provide the first genetic analysis of wolf-dog admixture in an area entirely recolonized, the northwestern Alps. As part of a long-term monitoring program, we performed genetic screening of thousands of non-invasive samples collected in Switzerland and adjacent territories since the return of the wolf in the mid-1990s. We identified a total of 115 individuals, only 2 of them showing significant signs of admixture stemming from past interbreeding with dogs, followed by backcrossing. This low rate of introgression (<2% accounting for all wolves ever detected over 1998–2017) parallels those from other European populations, especially in Western Europe (<7%). Despite potential hybridization with stray dogs, few founders and strong anthropogenic pressures, the genetic integrity of the Alpine population has remained intact throughout the entire recolonization process. In a context of widespread misinformation, this finding should reduce conflicts among the different actors involved and facilitate wolf conservation. Real-time genetic monitoring will be necessary to identify potential hybrids and support an effective management of this emblematic population.

## Introduction

Anthropogenic hybridization between wild species and their domesticated counterparts is a central concern in conservation biology. Introgression of artificially selected alleles may be beneficial, by bringing adaptive genetic variation into natural gene pools^1^. Alternatively, it may threaten wild populations due to genetic homogenization, incompatibilities and/or disruption of locally-adapted allelic combinations^2–5^. In any case, crossbreeding and subsequent introgression jeopardizes the genetic integrity of wild populations, on which legal actions are enforced by institutional stakeholders.

The grey wolf (*Canis lupus*) and its domesticated form, the dog (*C. l. familiaris*), make one of the most emblematic and complex example of this issue. After their nearly complete eradication in large parts of their original distribution over the last centuries, wolves are now recovering and recolonizing parts of their former ranges^6^, raising serious concerns regarding threats to livestock to the point that their management has become a political debate. Wolf-dog hybridization is a central aspect of this debate. Hybrids have been reported throughout Europe (Table 1), essentially from asymmetric crosses (male dogs × female wolves), with backcrossed individuals subsequently integrated into wolf packs^7–11^ (but see^12^). While wolf is under strict protection by several international treaties, although with national derogations to reduce predation and conflicts with local husbandry, crossbred individuals fall into a legal gap. Recommendations include their removal to protect the integrity of wild populations^13–16^. Hence, characterizing the genetic nature of expanding wolves is a major aspect of their conservation, even more so since morphological criteria potentially diagnostic of hybrids (i.e. black coat, white claws and spur on the hind legs) are unreliable to properly identify admixed individuals^17–19^.

**Table 1:** Summary of genetic surveys estimating population-based admixture rates in wild wolf samples, as well as confirming hybrids suspected from morphological, behavioral and/or preliminary genetic analyses.

Location	Period	Admixed/Confirmed	Resources	Remarks	Ref.
***Admixture estimation in wild populations***					
Italian Apennines (full range)	~1984–1999	1/107 (0.9%)	18 STRs	—	^7^
Italian Apennines (full range)	1987–2002	11/220 (5.0%)	16 linked-STRs	—	^9^
Italian Apennines (central-southern)	2000–2012	7/107 (6.5%)	18 STRs	—	^53^
Italian Apennines (northern)	2000–2009	16/430 (3.7%)	12 STRs + mtDNA CR + 4 Y-linked STRs	—	^68^
Italian Apennines (central, two areas)	2004–2013	14/47 (29.8%)	12 STRs + mtDNA CR + 2 Y-linked STRs	local hybridization events	^21^
Spain (north-western)	—	0/20 (0.0%)	13 STRs + mtDNA CR	—	^69^
Spain (north-western, one area)	2013	4/67 (6.0%)	18 STRs + mtDNA CR	three-months sampling	^36^
Spain + Portugal (full range)	1996–2009	8/212 (3.8%)	42 STRs + mtDNA CR + 6 Y-linked STRs	—	^11^
Portugal (central-western, one area)	2011–2014	1/21 (4.8%)	24 STRs + mtDNA CR	three packs studied	^70^
Georgia (full range)	2008–2012	14/102 (13.7%)	8 STRs + mtDNA CR	guarding dogs widespread	^22^
Bulgaria (full range) and Greece	2000–2011	10/102 (9.8%)	14 STRs + mtDNA CR	feral dogs widespread	^71^
Croatia (full range)	1996–2011	5/176 (2.8%)	12 STRs + mtDNA CR + 4 Y-linked STRs	hybrids restricted to anthropogenic areas	^19^
***Confirmation of presumptive hybrids***					
Italian Apennines	1996–2011	24/30	39 STRs		^17^
Italian Apennines	1992–2015	68/68	170 K SNPs		^18^
Italian Tuscany	1993–2001	3/3	18 STRs		^72^
Spain	2011	9/13	13 or 52 STRs + mtDNA CR		^54^
Latvia	1997–1999	12/12	16 STRs + mtDNA CR		^8^
Estonia and Latvia	2008–2009	8/8	11 STRs		^12^
Norway	1998–1999	1/1	18 STRs + mtDNA CR + 1 Y-linked STR		^10^
Iran	—	7/7	15 STRs		^73^

Admixture rates depend on the *Q* admixture threshold chosen, which varied between 0.8 and 0.95 across studies.

In the recent years, a wide array of studies have comprehensively monitored the rate and modality of wolf-dog hybridization in expanding European populations (summarized in Table 1), which appears to depend on their level of disturbance (see below), in link with the abundance of feral (returned to the wild state) and stray (free-ranging) dogs. Accordingly, admixture remains low in Western Europe (i.e. 0 to 6.5% across the Italian Apennines and Iberia, Table 1), being locally higher in anthropogenic environments where stray dogs are common^19,20^, and where residing packs are introgressed^21^. The figures are expectedly higher in eastern countries (10–14% in Georgia, Bulgaria and nearby Greece), where free-ranging large-size guarding dogs are an issue^22^. All studies, however, have focused on recovering populations that never faced full eradication. In contrast, the risk of crossbreeding with dogs is expected to be significantly higher in newly recolonized areas. This is firstly due to the low number of founders involved: hybridization in the initial stages of colonization could lead to widespread introgression as the population expands. Second, the areas where the wolf formerly vanished are usually human-dominated, with low acceptance by locals. These conditions, and notably the potentially high rate of poaching, may disrupt their social structure, in turn favoring crossbreeding with stray dogs. For instance, in Croatia and Italy, many hybrids are found near human settlements with a high rate of human-caused mortality^19,20^.

The Alpine wolf population remains a major gap in the European wolf-dog hybridization literature. Exterminated in the French and Swiss Alps in the late 19^th^ century, and in the Italian Alps during the early 20^th^ century, wolves have been re-establishing across this range from relict Italian Apennine pockets ever since the early 1990s^23–26^. Their formerly high mitochondrial DNA diversity has now essentially been replaced by a single Control Region (CR) haplotype (thereafter the Italian haplotype), diagnostic of these regions and never found in dogs nor wolves elsewhere^24,27–30^. This new population thus makes an ideal system to test whether hybridization with dogs had stronger genetic consequences in recolonized areas. Listed as “endangered” (based on data from 2007^31^,), the Alpine wolf population is constantly increasing, forming about 65 packs in the last update (2015–2016), mostly located in the Western parts between France and Italy^32^. In addition to the lack of suitable habitat and persecution, potential hybridization with dogs is considered as a major threat^33^. In Switzerland, only three established packs occur, but tens of itinerant individuals migrate throughout the country and neighboring regions. Wolf presence is highly controversial in this country: despite its strictly protected status, the species is under very stringent management through selective removal of stock-raiding individuals (when predation impact is considered too high), but is facing strong pressure by livestock breeders, hunters and local communities^34^, which conveys public rumors such as that wolf-dog hybrids are widespread. Hence, inferring the rate of wolf-dog introgressive hybridization in the Alpine population is also crucial for its future management and social perception.

In this study, we conducted a genetic survey of all the wolves detected in the Swiss and adjacent territories, as part of an ongoing non-invasive genetic monitoring since the early stages of the recolonization process started more than 20 years ago^24,25^. We genotyped thousands of non-invasive DNA samples and performed admixture analyses of multilocus genotypes of putative wolves and reference dogs, in order to estimate the rate of hybridization and admixture in the newly colonized Alpine population.

## Results

### Genetic screening

We sequenced the left domain of the hypervariable mitochondrial (mtDNA) Control Region (CR) in a total of 3,463 non-invasive unidentified samples and 23 tissues from dead wolves (359–360 bp or 235–236 bp, see Methods). A total of 1,645 samples could be assigned to the private Italian wolf haplotype (47.2%), 409 to typical dog haplotypes (*C. l. familiaris*; 11.7%), 709 to red foxes (*Vulpes vulpes*; 20.3%), 361 to other mammals (10.4%) and 362 (10.4%) could not be either PCR-amplified or sequenced (due to contamination by several taxa, e.g. saliva samples on preys). The 68 reference dogs did not carry the Italian wolf CR haplotype, but only typical dog ones (Table S2).

**Figure 1:**
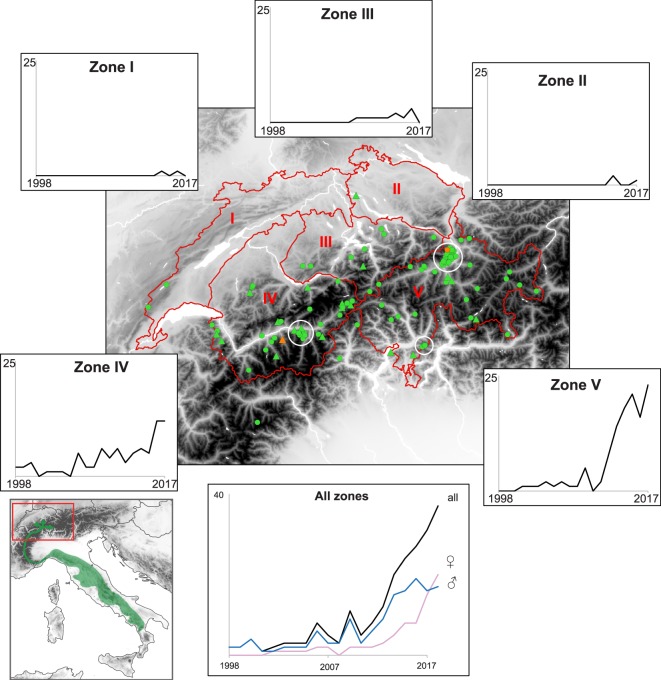
Evolution of the numbers of detected wolves in Switzerland in space and time. Place of last genetic detection (circles) or death (triangles) is shown on the map. Colors distinguish pure (green) from admixed wolves (orange). Approximate locations of the three residing packs are encircled in white. Wolf management compartments as defined by the FOEN are delineated in red. The bottom left panel frames the study area (red) and the main recolonization pathway presumably taken by the wolf from the Apennine population (green arrows) according to^24–26^.

Reliable microsatellite genotyping (11 loci) according to the multitube approach was successful in 874 out of 1,645 putative wolf samples (53%; scat: 60%; saliva: 50%; hair: 22%; urine: 22%; regurgitate: 100%; blood: 43%; tissue: 100%), identifying 115 different individuals from 1998 to 2017 (79 males, 36 females), with their number of detections ranging from 1 to 62 (average: 7.6; Table S1). Allelic dropout and false allele rate across loci averaged 5.8% and 0.9% respectively. The number of wolves increased over years, males first (Fig. 1 and Table S1). The maximum consecutive detection of a single individual reached at least seven years (2011–2017), and 21 wolves were ultimately found dead or legally removed from the population. An additional two died in Germany.

### Population genetic and admixture analyses

The Swiss wolves met Hardy-Weinberg Equilibrium (HWE) and appear panmictic among the three mainly occupied management compartments defined for Switzerland (n = 73, non-significant pairwise F_st_); note that this analysis excluded presumptive cubs from the three packs, known from field observations and confirmed by their genetic profiles. We found similar levels of diversity between these compartments (observed heterozygosity, H_e_ = 0.62–0.64; Allelic richness, A_r_ ~ 2.4, scaled on two individuals). This lack of genetic structure is also supported by the frequent movement of individuals between Swiss cantons (i.e. states; Table S1). The full wolf dataset had a probability of identity (PID) of 2.2 × 10^–8^ and a PID_sibs_ of 3.8 × 10^−4^.

**Figure 2:**
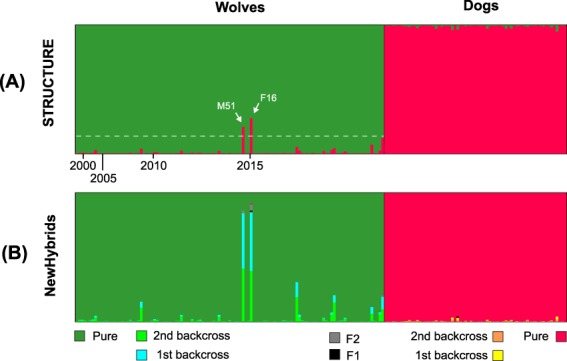
Bayesian clustering of individual wolf and dog genotypes (**A**) with STRUCTURE into two groups, and (**B**) with NewHybrids into eight genotype classes, including six hybrid and two parental classes (n = 99 pure wolves and 55 pure dogs). Individuals are arranged by their time of first detection. The dotted line show the threshold computed from analyses of simulated hybrids (Fig. S1). The two individuals above this threshold are shown.

**Figure 3:**
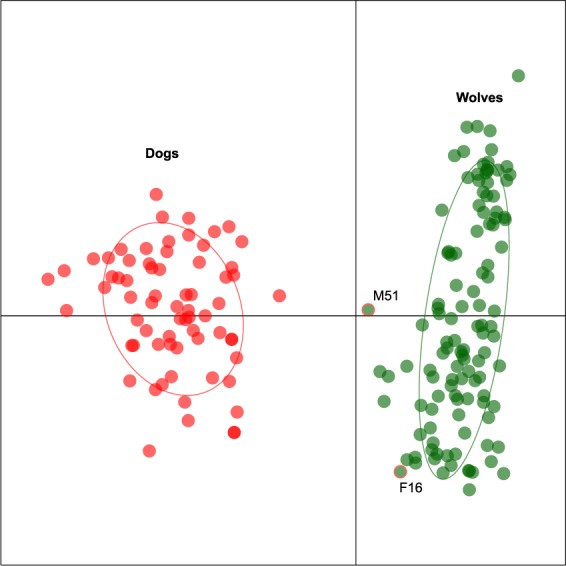
Principal Component Analysis (PCA) on individual wolf (green) and dog (red) genotypes. The two admixed wolves identified by the admixture analyses are shown by red frames. Ellipses correspond to the 80% inertia of each group.

Bayesian clustering analyses of the 115 putative wolves and 68 reference dogs (obtained from Switzerland) by STRUCTURE^35^ unambiguously discriminated between the two groups (K = 2, ΔK = 1485.0) (Fig. 2A). Principal Component Analysis (PCA) on individual genotypes also recover the two corresponding clusters along the first axis (Fig. 3). At least four individuals, however, were not perfectly assigned to their corresponding group, i.e. STRUCTURE admixture coefficients (*Q)* below 1.0 and intermediate positions on the first axis of the PCA (Fig. 3). One challenge is to interpret whether such pattern arises from recent gene flow or imperfect clustering due to shared polymorphism (i.e. false positive), which is bounded by the number and informativeness of the genetic marker used^19,36^. To address this issue, we simulated different classes of hybrids, using genotypes that were correctly assigned to their clusters (“pure” wolves and dogs, *Q* > 0.99) as parental references, on which we conducted similar analyses (Fig. S1). F1s, F2s and wolf first generation backcrosses received average wolf ancestry *Q*_*w*_ values of 0.48 (95% CI: 0.28–0.72), 0.50 (95% CI: 0.26–0.79), 0.73 (95% CI: 0.50–0.94) respectively, and thus marginally encompass those of pure individuals (95% CI: *Q*_*w*_ = 0.90–1.0; Fig. S1). However, the distinction becomes difficult with second-generation backcrosses (average *Q*_*w*_ = 0.89, 95% CI: 0.61–0.96; Fig. S1). Following the methodology of previous studies^19,36^, we used the minimum *Q*_*w*_ value of pure individuals (0.86) as an ad-hoc threshold to consider individuals as admixed.

From the empirical dataset, only two putative wolves (out of 115; 1.7%) were assigned with a probability lower than *Q*_*w*_ = 0.86 and hence were most likely introgressed by dogs (Fig. 2): individuals M51 (*Q*_*w*_ = 0.72; male, 9% of missing data) and F16 (*Q*_*w*_ = 0.79; female, no missing data). Inspection of the 90% confidence intervals for *Q*_*w*_ did not encompass 1.0 for M51 (0.40–0.96), confirming its admixed nature, but it did for F16 (0.54–1.0). Two additional individuals received low Q_w_ ( < 0.95), i.e. M83 (*Q*_*w*_ = 0.88; male), M80 (*Q*_*w*_ = 0.93; male), although these genotypes respectively featured 31% and 18% of missing data.

Moreover, Bayesian assignment of empirical genotypes to hybrid classes (F1, F2, first and second backcross generations in each direction) with NewHybrids^37^ confirmed that almost all Alpine wolves had the best probabilities to be purebred animals, except for M51 and F16, which shared an equal probability to be first or second generation backcrosses (Fig. 2B). Five additional individuals had cumulated probabilities above 0.1 to be backcrosses, although there were always primarily assigned to the parental wolf class, rather than to any other class.

## Discussion

Our long-term genetic monitoring supports very limited wolf-dog hybridization and introgression in the Swiss Alps and surroundings, confirming the genetic integrity of this recently established wolf population throughout time. Over the past two decades, no F1 hybrid was detected and only two wolves (out of 115) featured significant signs of introgression by dogs, likely stemming from backcrossing. There were accordingly assigned as first or second generation wolf backcrosses. We cannot exclude that other individuals descend from older hybridization events, with the little dog ancestry they hypothetically retained confounding with the standard variation shared by the two parental genomes at our limited set of loci. Because these crossbreeding events are very rare, the wolf gene pool is rapidly restored due to gene flow from pure individuals. While our resolution seems sufficient to detect the first backcrosses (as in other studies, Table 1), false positives could arise if individual genotypes are uninformative or incomplete^17,19^. Here, the two individuals M80 and M83, with Q_w_ < 0.95, were among the very few with > 15% of missing data, and hence unreliable estimates (Table S1 and Methods).

The anecdotal rate of admixture in the Alpine wolves ever since their very first establishment (<2% considering all individuals since 1998) is in line with other European populations, although these were mainly studied over shorter timeframes (Table 1). It is also supported from a recent assessment in the Italian Alps, which did not identify any hybrid (www.lifewolfalps.eu). Feral and stray dogs are supposedly absent in Switzerland, and wolves usually avoid the immediate vicinity of human settlements. All dogs detected here are doubtlessly pets, hunting or guarding dogs, the Swiss mountains being extensively hiked and exploited for pastoralism. Although local crossbreeding cannot be ruled out, these rare hybridization events may have rather occurred in the Apennines, the source population of Alpine wolves^24,25^. About a million of free-ranging dogs has been reported over Italy by perhaps outdated estimates (10% of feral individuals^38,39^). While the overall dog introgression rate of Italian wolves still remains low (<7%, Table 1), some localized areas host introgressed packs^21^. Among them, western Tuscany, at the northwestern edge of the Apennine population, appears to be a hotspot of wolf × dog hybridization^20^, near the corridor connecting the Apennines and the Alps. Alpine wolves thus seems to have avoided gene flow with dogs and admixed individuals, which is remarkable given the low numbers of effective founders that arrived in this human-dominated region (~15^25^). The high mortality rate in Switzerland (21 out of the 115 individuals analyzed) could have also hampered the rapid establishment of stable social structures, promoting mating with dogs instead (as shown in wolf-coyote hybridization^40^); the first Swiss pack settled >16 years after the species’ arrival. Therefore, and despite these suboptimal initial conditions, the now established Alpine population has so far resisted the risks of hybridization.

Note that because we identified putative wolves from non-invasive samples based on maternally transmitted mtDNA (i.e. the diagnostic CR Italian haplotype), we only tested admixture resulting from crossbreeding between female wolves and male dogs. Accordingly, all wolf genetic surveys from Italy and the Alps published to date (featuring thousands of samples) failed to detect the reverse (dog mitochondrial introgression in wolf populations; reviewed in^41^). Potential reasons for this asymmetry are multiple, e.g. less aggressiveness of female wolves towards dogs (while male wolves prey on them), longer partner-seeking activity by female wolves, continuous physiological availability of male dogs for mating, greater survival chances of pups raised by wolf mothers in the wild (^12^ and references therein). Accordingly, all tissues from dead wolves identified here featured the Italian CR haplotype (n = 23). As populations are reconnecting, other wolf haplotypes have recently been found in the eastern Alps (originating from the Balkans and Central Europe^42,43^) and even southern Italy^44,45^, but so far have not reached our study area^30,41^. The absence of nuclear differentiation between Swiss wolves also confirms that the recolonization of the Alpine arc stems from a single source, as previously suggested^24,25^. Moreover, it indicates that the environmental conditions in Switzerland are favorable enough that itinerant and resident wolves remain panmictic, implying a good connectivity, as also suggested by the migration patterns of individuals between cantons (Table S1).

The idea sometimes propagated that Alpine wolves are “hybrids”, largely based on unconvincing and unpublished evidence, is thus not supported by our genetic data. This misconception is confusing the public debate between the various actors involved, e.g. livestock breeders, hunters and institutional stakeholders. Wolves and dogs obviously share a common evolutionary history, intertwined by recurrent crossbreeding ever since the earliest stages of domestication, between 35,000 and 11,000 years ago^46^. As a result, ancient dog breeds feature traces of admixture with wolves and, reciprocally, most Eurasian wolf populations acquired dog alleles through past introgression events, which can confound with ancestral polymorphisms maintained across these recently diverged taxa^47–49^. While this should not be confused with contemporary hybridization, it conditions why detecting wolf-dog hybrids for management purposes is far from trivial, given the limitation of resources (i.e. number and nature of analyzable genetic loci) imposed by genetic profiling of wild individuals from low-quality non-invasive samples. New methodologies and technologies (e.g. high-throughput microsatellite genotyping^50^; SNPs^51,52^) will allow better resolution to detect hybrid backcrosses from pure individuals in future genetic screening.

In Switzerland, the recolonizing wolves have thus retained their genetic integrity. The two confirmed backcrossed individuals are no longer in the country. Female F16 arrived and settled in the Central Alps from June 2014 to February 2017, when it was ultimately poached (Table S1). Male M51 was only detected at nine instances from February to August 2015 in the eastern and southern parts of the country. None of the parents, and by extension their offspring, of the three resident wolf packs feature signs of dog introgression. In order to protect this population, we stress the need to prevent dog vagrancy in the species’ expansion range, as well as to legally remove any F1 hybrids as soon as they are detected by real-time genetic screening and/or suspicion from morphological characters. In the presumed absence of dogs, these F1 hybrids, potentially migrating from Italy, are the proximate cause of subsequent dog introgression into the wolf gene pool, and should be the main target of legal regulations. In contrast, managing admixed individuals beyond F1s seems neither relevant nor efficient, since the wolf genome is being restored, and because effectively tracing such level of admixture remains challenging. External criteria are indeed unreliable to identify backcrosses, even from dead animals: despite its dog introgression, no question was raised regarding female F16. Reciprocally, this questions the validity of so-called “reference” wolves identified by morphology; putative wolves should only be selected based on molecular analyses (e.g.^21,36,53,54^, this study). Finally, we emphasize the crucial role played by such genetic monitoring, which provides an empirical basis to move the public debate forward, and hopefully improve the tense relationship between the many actors discussing the fate of large predators claiming back their former ranges.

## Methods

### Study area and sample collection

The study was conducted in Switzerland and neighboring territories (nearby France and Italy), covering an area of about 45,000 km^2^, as a part of a long-term non-invasive genetic monitoring of the species since its recolonization of the Alpine range in the 1990s. A total of 3,486 samples were analyzed. These include 3,463 unidentified samples, comprising 1,191 scats (34.4%), 2,007 saliva swabs collected on preys (58.0%), 106 hair (3.1%), 119 urine samples (3.4%), 7 regurgitates (0.2%) and 33 blood samples (0.9%), which were non-invasively collected in the field from November 1998 to December 2017 by trained rangers and wardens from Swiss regional wildlife offices and researchers. In addition, tissues from 23 dead wolves (accidentally, illegally or legally killed) were also analyzed. The geographic origin of samples and their number of detections are given in Table S1. Sites for non-invasive sampling were chosen opportunistically based on known or presumed wolf presence, documented prey kills or random direct observations. Tissue, scat and hair samples were stored in 80–95% ethanol at +4 or −20 °C. Swab samples were stored dried and immediately processed after arrival in the laboratory. Regurgitate and urine samples were stored at −20 °C.

No ethics approval was necessary to work with non-invasive samples or tissues from dead animals. Fieldwork procedures were specifically approved by regional wildlife offices and the Federal Office for the Environment (FOEN) as a part of national wolf monitoring activities.

### DNA extraction, sequencing and genotyping

DNA from scat samples was extracted with the QIAamp® DNA Stool or Fast DNA Stool Mini kit (Qiagen), while all other samples except urine were extracted with the QIAamp® Tissue or DNeasy® Blood & Tissue kit (Qiagen), following manufacturer’s instructions. DNA from urine samples collected in snow was extracted according to^55^. Species identification was assessed by sequencing a 359–360 bp portion of the left domain of the mtDNA Control Region (CR; primers and methods in^24^), except for saliva samples for which we used a newly-designed ungulate-unspecific internal primer (HW3: 5′-GCCCTTATTGGACTAAGTG-3′; 235–236 bp amplified) in order to avoid co-amplification of undesirable prey DNA. DNA sequencing was performed on an ABI3100 platform (Applied Biosystems), after purification with the QIAquick PCR Purification kit (Qiagen) or Wizard® SV Gel and PCR Clean-Up System (Promega). All DNA extractions and pre-PCR setups occurred in a physically-separated laboratory exclusively devoted to the analysis of low copy number DNA samples. Negative controls were employed during all extraction and amplification experiments to monitor contaminations.

Eleven microsatellite (i.e. STR) loci (FH2054, FH2140, FH2161, FH2096, FH2137, FH2088, FH2001, FH2010^56^, PEZ17, PEZ1^57^, CPH5^58^) and a Y-chromosome sex marker (Sryw f: 5′- GCCGAGTCCTCTCCTGTA-3′/Sryw r: 5′-TTGTATGAACCATCATTGTGA-3′) were amplified, following the multiplex preamplification method^59^, with some modifications. The principle of this two-step PCR approach is that an initial large-volume PCR is carried out including primers for all 12 markers to be genotyped. A post-amplification aliquot from this PCR is then used as template in separate amplifications for each marker to genotype individuals. This procedure was repeated eight times (four times for tissues) to obtain a consensus genotype, following the multi-tube approach^60^. Multiplex amplifications were performed in 50 µl reactions containing 2.5 mM MgCl2, 0.1 mM of each dNTP, 0.01 µmol of each primer, 0.2 mg/ml BSA, 1 x PCR Buffer and 1 U of AmpliTaq® Gold DNA polymerase (Applied Biosystems), and 20 µl of extract. For multiplex reactions we used an annealing temperature of 50–55 °C and 25 PCR cycles. The second PCR step was performed in 20 µl reactions containing 2.5–5.0 mM MgCl_2_, 0.1 mM of each dNTP, 0.2–0.5 µmol of each primer, 0.2 mg/ml BSA, 1 x PCR Buffer and 1 U of AmpliTaq® Gold DNA polymerase (Applied Biosystems), and 2.0 µl of extract. We used an annealing temperature of 50–57 °C and 40 PCR cycles. One primer of each pair was synthesized with a 5′-end fluorescent dye (ATTO532, ATTO550, Dyomics 630, FAM and HEX) to allow detection and sizing of fragments on an ABI Prism 3100 DNA sequencer (Applied Biosystems). Alleles were scored using GeneMapper 4.0 (Applied Biosystems). Consensus genotypes, allelic dropout and rates of false alleles were computed manually as follows: heterozygous at one locus were accepted if both alleles appeared at least four times among the eight replicates, and homozygous were accepted if the genotype was observed in five independent PCRs. If neither of those cases occurred, the alleles were treated as missing data (NA). In addition, we genotyped saliva samples of dogs (n = 68) from 35 different breeds, obtained in Switzerland through veterinary practice, to be used as a reference (Table S2). The final dataset (n = 183) included 131 individuals with no NA (72%), 41 with 0–10% NA (22%), 7 with 10–20% NA (4%), and 4 with 20–45% NA (2%) (Details in Tables S1 and S2).

### Standard Genetic Analyses

We assigned individuals to the management compartments defined by the FOEN for this species (Fig. 1), based on the area they spent most of their time according to our genetic detections. Individuals sampled in neighboring France and Italy where assigned to the closest compartment. For the three main occupied compartments (n = 7, 35 and 31 for compartments III, IV and V, respectively, not-including cubs from packs) we computed observed heterozygosity (H_e_), allelic richness (A_r_), pairwise F_st_ (with significance tested by 1,000 permutations), and performed test for Hardy-Weinberg Equilibrium (HWE) in FSTAT^61^. Probabilities of identity (PID) were estimated with GenAlEx 6.5^62^.

### Admixture analyses

We assigned putative wolves (n = 115) and dogs (n = 68) to groups with the Bayesian clustering algorithm of STRUCTURE^35^. We used the admixture model and ran 10 replicates for K from 1 to 11, each including 100′000 iterations after a burnin of 10′000. Replicates were combined with CLUMP^63^ and admixture proportions *Q* were visualized with DISTRUCT^64^. The ΔK statistic^65^ was computed to infer the number of K best explaining the data (STRUCTURE HARVESTER^66^). In parallel, we conducted a PCA to assess the genetic variance between individuals (*ade4* and *adegenet* R package^67^).

In order to infer the nature of individuals that featured intermediate STRUCTURE *Q* values, we used the software NewHybrids^37^ to compute their probability of belonging to the six following hybrid classes: F1, F2, wolf first generation backcross, wolf second generation backcross, dog first generation backcross and dog second generation backcross. We pre-assigned pure individuals (*Q* > 0.99 in the STRUCTURE analyses) as parental references (n = 99 wolves and 55 dogs). The chain was ran for 100′000 iterations, which was sufficient to reach stationarity.

We further implemented a simulation approach to evaluate the power to discriminate between pure and introgressed individuals from our dataset. To this end, we generated F1, F2 and backcrossed hybrids (100 for each category) from the reference individuals selected above, using the *hybridize* function of *adegenet*. These simulated and pure genotypes were then analyzed by STRUCTURE (K = 2) and PCA, in the same way as the empirical dataset (see above). This allowed to obtained the distribution of *Q*_*w*_ (the wolf ancestry of individual) for each hybrid class, and calculate the threshold below which individuals can be considered admixed without confusion with pure ones^19,36^.

## Supplementary information

Supplementary Information

## Data Availability

Microsatellite genotypes are available from the Dryad Digital Repository (10.5061/dryad.7g2g68d).
