# Supplementary material for: Two decades of non-invasive genetic monitoring of the grey wolves recolonizing the Alps support very limited dog introgression
Source: Sci Rep. 2019 Jan 16;9:148. doi: 10.1038/s41598-018-37331-x (PMC6335406; doi:10.1038/s41598-018-37331-x)
Supplement: Supplementary file 1 — Supplementary Information [file 41598_2018_37331_MOESM1_ESM.pdf]

Supplementary Information for:

Two decades of non-invasive genetic monitoring of the grey wolves recolonizing the Alps support very limited dog introgression

Christophe Dufresnes[1,2], Nadège Remollino[1], Céline Stoffel[1], Ralph Manz[3], Jean-Marc Weber[4] & Luca Fumagalli[1*]

[1] Laboratory for Conservation Biology, Department of Ecology and Evolution, Biophore Building, University of Lausanne, CH-1015 Lausanne, Switzerland.

[2] Department of Animal & Plant Sciences, University of Sheffield, Alfred Denny Building, Western Bank, Sheffield S10 2TN, United Kingdom.

[3] KORA, Carnivore Ecology and Wildlife Management, Thunstrasse 31, CH-3074 Muri, Switzerland.

[4] Fauna Découverte, Pré-Girard 18, CH-2067 Chaumont, Switzerland.

*Corresponding author

luca.fumagalli@unil.ch

Table S1: Details on the 115 wolves detected in this study. Numbers correspond to detections throughout the years, colored by sex (blue: males, pink: females), †: individual found dead or legally removed. Introgressed individuals are highlighted in red (see remarks). tot. detect.: total number of detections; % NA: percentage of missing genotypic data; manag. comp.: management compartment (see Fig. 1) where the individual spent much of its time (according to detections); cantons / area: F: nearby France; I: nearby Italy; BE: Bern; GL: Glaris; GR: Graubünden; FR: Fribourg; LU: Luzern; NW: Nidwald; OW: Obwald; SG: St. Gallen; SZ: Schwyz; TG: Thurgau; TI: Ticino; UR: Uri; VD: Vaud; VS: Valais; ZH: Zürich. Brackets indicate neighboring areas for individuals close to borders.

	1998	1999	2000	2001	2002	2003	2004	2005	2006	2007	2008	2009	2010	2011	2012	2013	2014	2015	2016	2017	tot. detect.	% NA	cantons / area	manag. comp.	remarks
**M01**	1†																				1	0	VS	IV	
**M02**	1	1†																			2	0	VS	IV	
**M03**		4	3																		7	0	VS	IV	
**M04**			2																		2	0	VS	IV	
**M06**			1†																		1	0	VS	IV	
**M07**			4	1†																	5	0	I(TI),GR	V	
**F01**					1	9	2	1													13	4	VS, I(VS)	IV	
**M09**					1	2	1		1												5	0	GR	V	
**M10**						4															4	0	F	I	
**M11**							2	1	1			1		3	2						10	0	TI	V	
**M12**								1													1	4	GR	V	
**M13**									1†												1	0	BE	IV	
**M14**									2												2	0	I(VS)	IV	
**F02**									1	4											5	0	VS	IV	
**F03**									1†												1	0	VS	IV	
**M15**									1†												1	0	VS	IV	
**M16**									1	11	8	9	3†								32	0	BE,FR,VD,VS	IV	
**F04**										1											1	0	I(VS)	IV	
**M17**										1											1	4	I(VS)	IV	
**M18**											1										1	9	TI	V	
**M19**										1		1									2	4	GR	V	
**M20**											3	10	5	7	10						35	0	VS,OW,LU,BE	III	
**M21**												12†									12	0	VS	IV	
**M22**												1									1	9	GR	V	
**F05**												9	13	28	8	4					62	0	BE,FR	IV	
**F06**												15	1								16	0	VS	IV	
**M23**												1									1	18	VS	IV	
**M24**												4									4	0	VS,GR	V	
**M25**												1									1	4	GR	V	
**M27**													1								1	45	VS	IV	
**M28**														13	4	7	5				29	0	TI,VS,I(VS)	IV	
**F07**														4	4	24	7	5	2	5	51	0	VS,SG,GR	V	Parent of pack GR
**M29**														5							5	0	VS	IV	

**M30**	4	7	22	8	6	3	4	54	0	VS,SG,GR	V	Parent of pack GR
**M31**	2							2	0	VS	IV	
**M32**		1	1	5				7	0	TI,GR	V	
**M33**		1	5					6	4	GR	V	cub of pack GR
**M34**		1	25					26	4	GR,VS	IV	cub of pack GR
**F08**		2			3	3	1	9	0	TI	V	parent of pack TI
**M35**		7	17†					24	4	VS	IV	
**M36**		1	9†					10	4	GR,TI	V	cub of pack GR
**M37**			1					1	13	GR	V	cub of pack GR
**M38**			11	2				13	0	GR,VS,VD	IV	cub of pack GR
**M39**			1					1	9	I(F)	-	
**F09**			1					1	9	I(F)	-	
**M40**			4					4	0	UR	III	
**M41**			2	1				3	4	TI,GR	V	
**M42**			1	1†				2	0	GR	V	cub of pack GR
**M43**			2	11†				13	4	GR,SG,GL,SZ,ZH	V	cub of pack GR
**F10**			1	4				5	4	GR	V	cub of pack GR
**F11**			1	17	1	1	2	22	0	GR	V	cub of pack GR
**M44**			2	1†				3	0	TI,GR	V	
**F12**				4				4	4	GR	V	cub of pack GR
**M45**				9				9	0	OW,SZ,SG,AR,VS	III	
**F13**				9	4	5	2†	20	4	FR,BE	IV	
**M46**				7				7	4	VS	IV	
**F14**				3	7	9		19	0	VS	IV	parent of pack VS
**M47**				1	5	4	5	15	0	TI,GR	V	parent of pack TI
**M48**				1	3†			4	0	GR	V	cub of pack GR
**M49**				1	1			2	4	GR	V	cub of pack GR
**M50**				2	3			5	0	GR	V	cub of pack GR
**M51**					9			9	9	TI,GR,SG	V	backcrossed wolf-dog hybrid
**M52**					13	3	2	18	0	GR,SG,SZ	V	cub of pack GR
**M53**					7†			7	4	GR	V	cub of pack GR
**M55**					3			3	0	GR,SG	V	cub of pack GR
**F15**				1	1†			2	4	GR,TI	V	cub of pack GR
**M56**					3	2		5	0	SG,GR	V	
**M57**					2			2	9	TI,GR	V	
**M58**					5			5	0	OW,NW	III	
**M59**					9	12	7	28	0	VS	IV	parent of pack VS
**F16**				3	4	10	1†	18	0	VS	IV	backcrossed wolf-dog hybrid
**F17**					2	3		5	13	GR,SG	V	cub of pack GR
**M60**					2			2	0	GR	V	cub of pack GR
**M61**					2	1	2	5	0	TI,GR	V	
**M62**					5	2		7	0	GR,SG,VS	V	cub of pack GR
**M63**					1	2†		3	4	VS	IV	

**M64**			11	1	12	9	VS,FR,BE	IV	
**M65**	2				2	9	GR,SG	V	cub of pack GR
**M66**		2			2	0	GR	V	cub of pack GR
**M67**		1	1†		2	0	GR	V	cub of pack GR
**F18**			6	11	17	0	GR	V	cub of pack GR
**F19**			3		3	0	VD	I	
**M68**			8†		8	0	UR,NW,OW	III	
**M69**	2				2	0	GR	V	cub of pack GR
**F20**			1	1	2	9	TI	V	cub of pack TI
**M70**			3		3	0	GR	V	
**F21**			1		1	0	GR	V	cub of pack GR
**F22**			4†		4	0	VS	IV	cub of pack VS
**M71**			2	4	6	0	GL,GR,SG	III	
**M72**			1		1	0	VS	IV	cub of pack VS
**F23**			1	2	3	0	VS	IV	cub of pack VS
**F24**			2	3	5	0	VS	IV	cub of pack VS
**M73**			3	10	13	0	VS	IV	
**M74**				9	9	0	BE,OW,NW	III	
**M75**				10	10	0	GR,TI,TG,ZH	V	
**M76**				8	8	0	GR,BE	V	cub of pack GR
**M77**			2		2	0	GR	V	cub of pack GR
**F25**			1		1	4	GR	V	cub of pack GR
**F26**				1	1	9	TI	V	cub of pack TI
**M78**				1	1	13	TI	V	cub of pack TI
**F27**				1	1	9	GR	V	
**F28**				5	5	0	VS,BE	IV	
**F29**				3	3	0	GR	V	
**F30**				1	1	4	GR	V	
**F31**				1	1	0	GR	V	
**F32**				4	4	0	GR	V	
**F33**				3	3	4	GR	V	cub of pack GR
**F34**				1	1	9	TI	V	
**F35**				3	3	0	SG	V	
**M79**				2	2	13	BE,SZ	IV	
**M80**				1	1	18	BE	IV	$Q_w < 0.95$ but 18% of NA
**F36**				1	1	0	GR	V	
**M81**				1	1	0	TI	V	cub of pack TI
**M82**				2	2	0	VS	IV	
**M83**				1	1	31	TI	V	$Q_w < 0.95$ but 31% of NA

Table S2: Information on the dog reference individuals used in this study. All featured dog CR haplotypes (*Canis lupus familiaris*); because hundreds of identical sequences are present on GenBank, we report the first BLAST hit, which was always 100% identical to the haplotype. % of missing microsatellite data is indicated

ID	Name	Breed	D-loop First Hit	% NA
CCH02	Bambou	Bulldog	MG818369	0
CCH03	Sam	Yorkshire Terrier	MH105046	0
CCH04	Isha	Cocker Spaniel	MG799266	0
CCH05	Solo	English Cocker Spaniel	MG818368	0
CCH06	Zara	German Shepherd	MG818368	0
CCH08	Gribouille	Yorkshire Terrier	MG818367	0
CCH09	Ocean	Border Collie	MG818363	9
CCH10	Mushu	Maltese	MG818367	0
CCH11	Oskar	Westie	MG818370	0
CCH12	Cheridan	Scottish Terrier	MG818368	0
CCH13	Basile	Rottweiler	MH105046	0
CCH14	Luna	Golden Retriever	MG818372	0
CCH15	Hamish	Westie	AY928921	0
CCH16	Spike	Pug	MG818367	0
CCH17	Jimony	Jack Russel Terrier	bad sequence	9
CCH18	Rocky	Labrador	MG818367	27
CCH19	Diablo	Pitbull	MG818370	0
CCH20	Mitzouko	Chihuahua	KX379528	0
CCH21	Joe	Cairn Terrier	MG818372	0
CCH22	Bambou	Poodle	MH105046	0
CCH23	Spring Roll	Jack Russel Terrier	MG818363	0
CCH24	Lana	Labrador	MG818367	0
CCH25	Milky	Labrador	MG818368	0
CCH26	Looping	Maltese	KJ637105	0
CCH27	Zita	Fox Terrier	MG818369	0
CCH28	Lucy	Pug	MG818370	0
CCH30	Lea	Appenzell Mountain Dog	MG818372	0
CCH31	Fila	Coton de Tulear	MG920436	0
CCH32	Sina	Spitz	HQ452459	0
CCH33	Glena	Yorkie Bichon	MH105046	0
CCH34	Jamie	Jack Russel Terrier	MG818368	0
CCH35	Candy	Yorkshire Terrier	MH105046	0
CCH36	Filou	Terrier	MG799266	0
CCH37	Filou	Bichon Frise	MG818367	0
CCH38	Sabrina	Wheaten terrier	MG818372	0
CCH39	Billy	Coton de Tulear	MG818372	0
CCH40	Fani	Cocker Spaniel	MG818368	9
CCH41	Sisi	Pekingese	MG818368	0
CCH42	Youpi	Yorkshire terrier	MG818372	0
CCH43	Snoopy	Border Collie	MG818369	9
CCH44	Jimmy	Jack Russel Terrier	MG818370	9
CCH45	Kali	Labrador	MG818367	0
CCH46	-	Pekingese	MG818372	0
CCH47	Scouby	Appenzell Mountain Dog	KY549991	0
CCH48	Douojy	Golden Retriever	MG818372	36
CCH49	Bambou	Poodle	MH105046	0
CCH50	Nala	Groenendael Dog	MG818367	0
CCH51	Patou	Appenzell Mountain Dog	KY549991	0
CCH52	JB	Yorkshire terrier	MG818372	0
CCH53	Oskar	West Highland White Terrier	MG818370	0
CCH54	Sally	Shetland Sheepdog	MG818367	0
CCH55	Timur	Yorkshire terrier	MH105046	0
CCH56	Akko	Bearded Collie	MH105046	0
CCH57	Praline	Jack Russel Terrier	MG818372	0
CCH58	Benje	Fox Terrier	MG818363	0
CCH59	Kaï	Chinese Crested Dog	KU647393	0
CCH60	Raïa	Miniature Dachshund Dog	MG818367	18
CCH61	Funny	Cavalier King Charles Spaniel	MG818367	0
CCH62	Orion	Cocker Spaniel	MH105046	0
CCH63	Janine	Papillon Dog	MG818369	0
CCH64	Cheyenne	Papillon Dog	MG818369	0
CCH65	Prunelle	Papillon Dog	MG818369	0
CCH66	Fanny	Rottweiler	MH105046	0
CCH67	Chelsa	Yorkshire Terrier	MG818372	0
CCH68	Brandy	Labrador	MH105046	0
CCH69	Cheyenne	Shetland Sheepdog	MG818370	0
CCH70	-	-	KY549991	9
CCH71	Kheira	Bernese Mountain Dog	KY549991	9

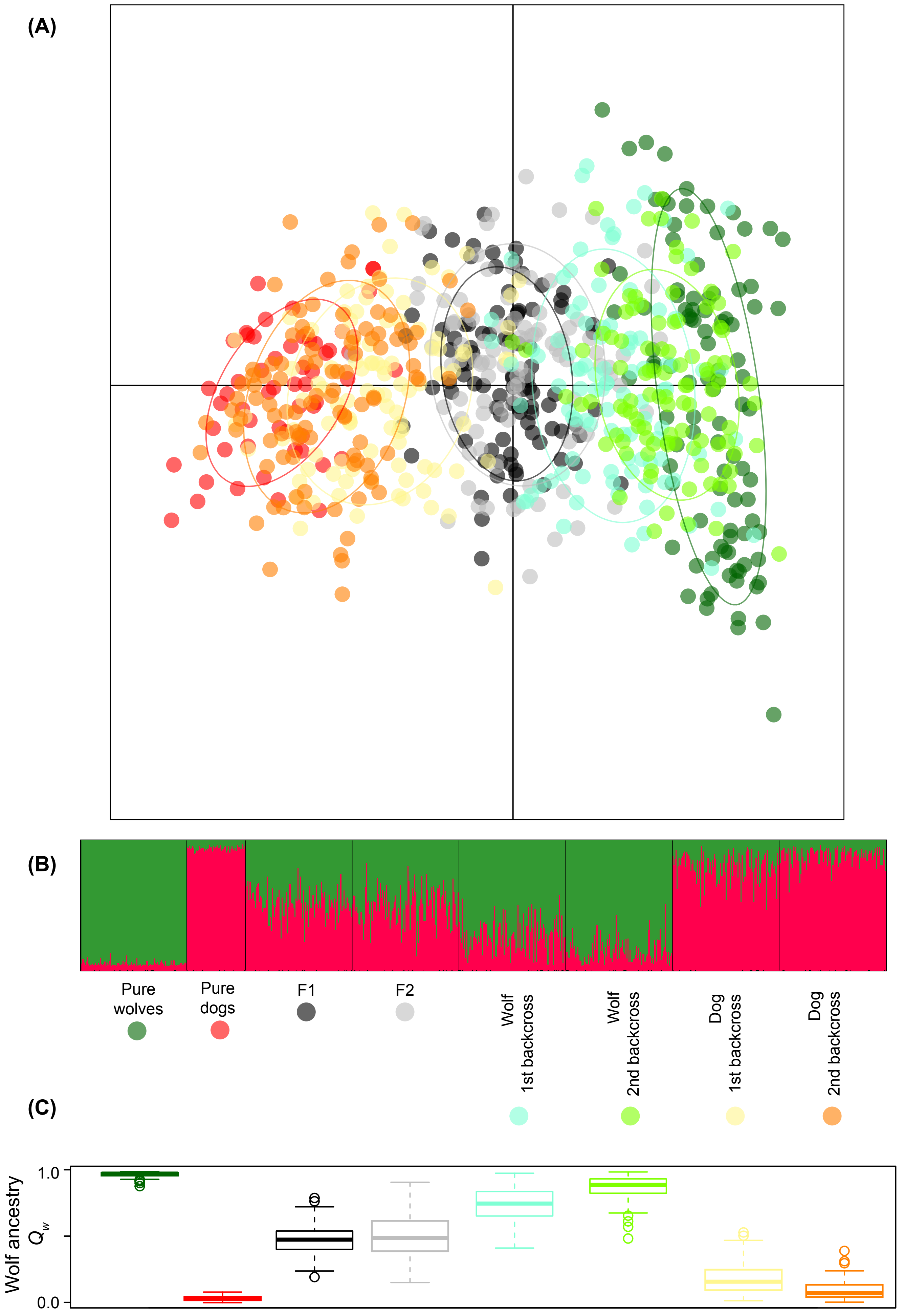

Fig. S1: Analyses of simulated hybrid genotypes with pure wolf and dogs individuals. (A) PCA, (B) STRUCTURE analysis; (C) distribution of the wolf ancestry estimated from STRUCTURE for each hybrid class. Color codes as follow: green: pure wolves, red: pure dogs, black: F1, grey: F2, light blue and green: 1st and 2nd generations wolf backcrosses, yellow and orange: 1st and 2nd generations dog backcrosses.
